# Supplementary material for: Preparation of Mechanically Stable Superamphiphobic Coatings via Combining Phase Separation of Adhesive and Fluorinated SiO2 for Anti-Icing
Source: Nanomaterials (Basel). 2023 Jun 16;13(12):1872. doi: 10.3390/nano13121872 (PMC10304342; doi:10.3390/nano13121872)
Supplement: Supplementary file 1 [file nanomaterials-13-01872-s001.zip › nanomaterials-2438589 supplementary materials.pdf]

Supporting Information

Preparation of Mechanically Stable Superamphiphobic Coatings via Combining Phase Separation of Adhesive and Fluorinated SiO_2 for Anti-icing

Jinfei Wei [1,2], Weidong Liang [1,*], and Junping Zhang [2,*]

[1] Department of Chemical Engineering, College of Petrochemical Engineering, Lanzhou University of Technology, Lanzhou 730050, China

[2] Center of Eco-material and Green Chemistry, Lanzhou Institute of Chemical Physics, Chinese Academy of Sciences, Lanzhou 730000, China

*Corresponding authors: wdliangh@lut.cn (W.L.); jpzhang@licp.cas.cn (J.Z.)

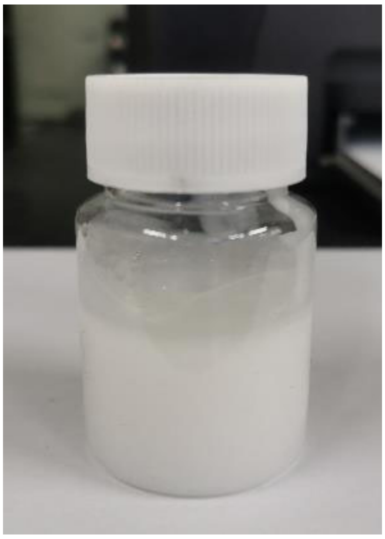

Figure S1. Photograph of the SPET/FD-POS@SiO_2 dispersion liquid. $V_{non\text{-}solvent}$ = 1.7 mL, m_{SPET} = 2.4 g.

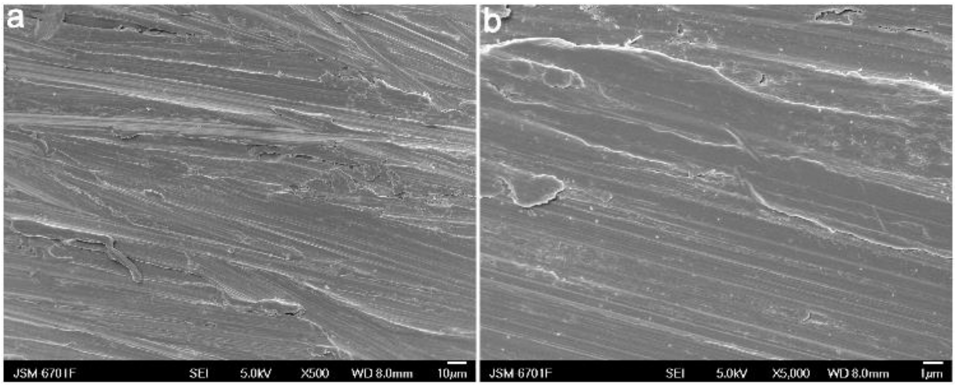

Figure S2. SEM images of the Al alloy plate.

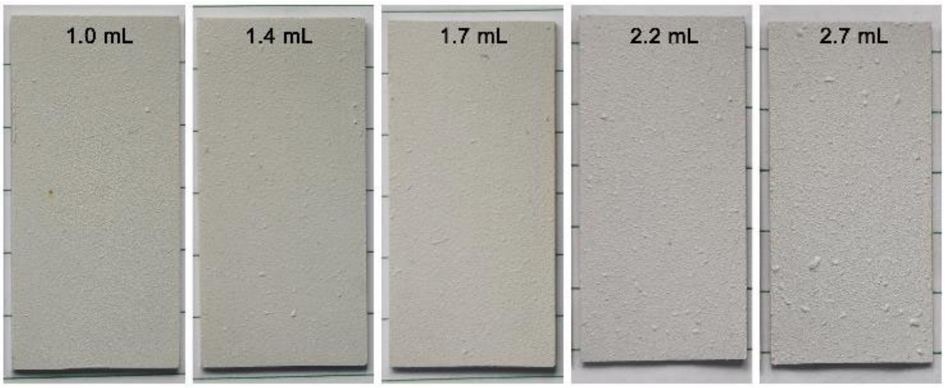

Figure S3. Photographs of the SPET/FD-POS@SiO_2 coatings with different non-solvent content. m_{SPET} = 3.0 g.

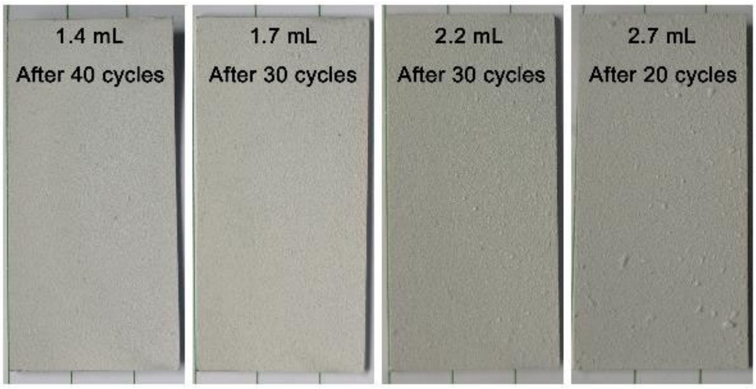

Figure S4. Photographs of the SPET/FD-POS@SiO_2 coatings with different non-solvent content after Taber abrasion test. m_{SPET} = 3.0 g.

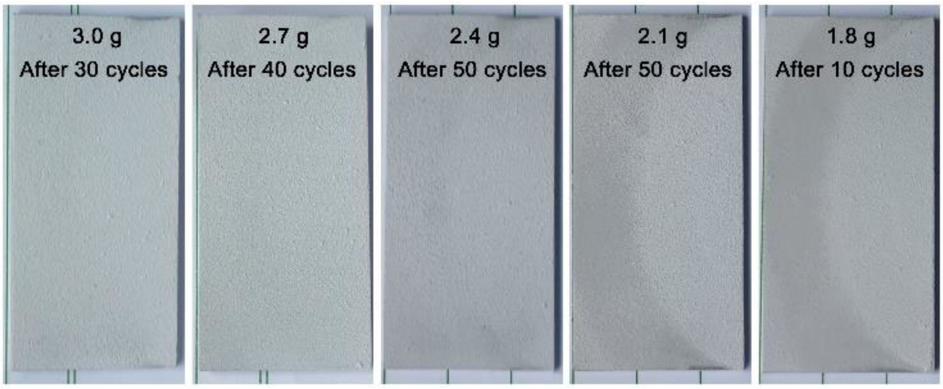

Figure S5. Photographs of SPET/FD-POS@SiO_2 coatings with different SPET amount after Taber abrasion test. $V_{non\text{-}solvent}$ = 1.7 mL.

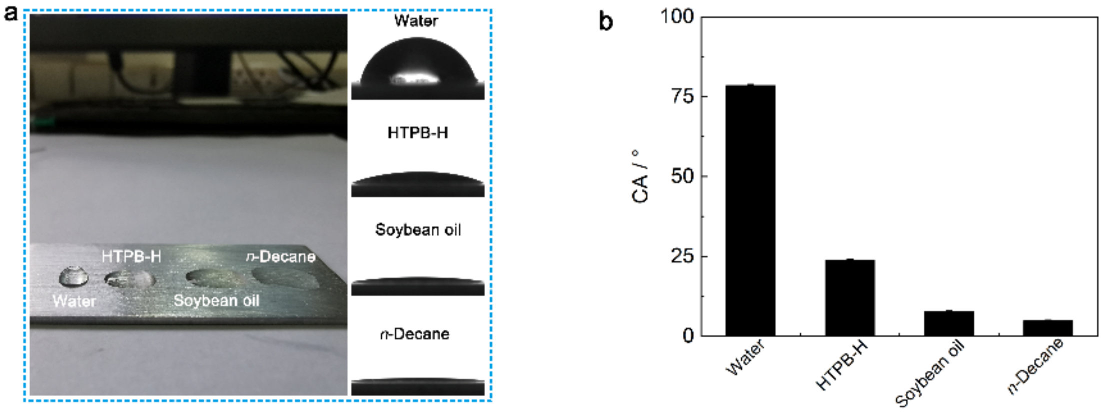

Figure S6. (a) Photographs of the Al alloy plate with droplets of different surface tension on the surface. (b) CA of various liquids on the Al alloy plate.

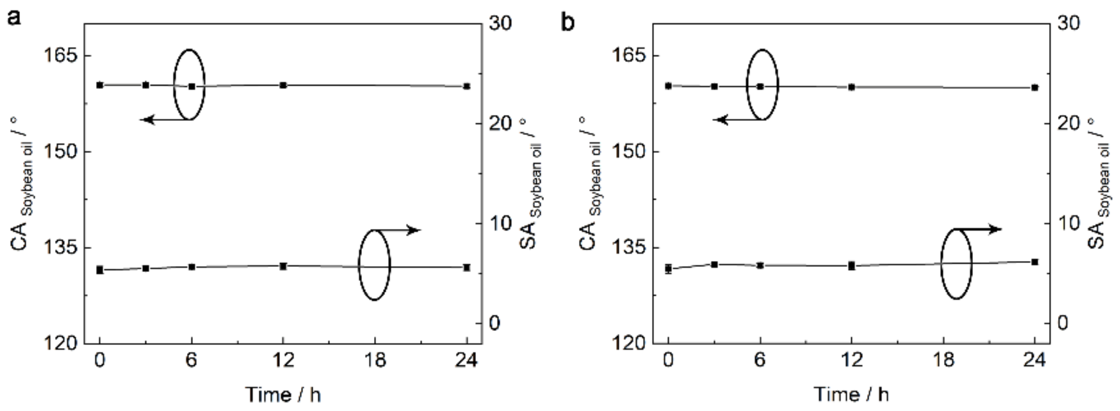

Figure S7. Changes of $CA_{soybean\ oil}$ and $SA_{soybean\ oil}$ of the SPET/FD-POS@SiO_2 coating during immersion in (a) water and (b) soybean oil. $V_{non\text{-}solvent}$ = 1.7 mL, m_{SPET} = 2.4 g.

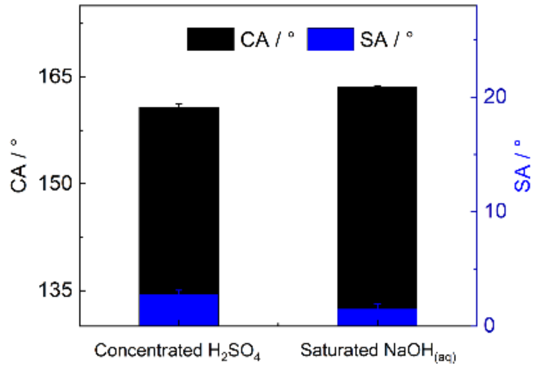

Figure S8. CA and SA of concentrated H_2SO_4 and saturated $NaOH_{(aq)}$ on the SPET/FD-POS@SiO_2 coating. $V_{non\text{-}solvent}$ = 1.7 mL, m_{SPET} = 2.4g.

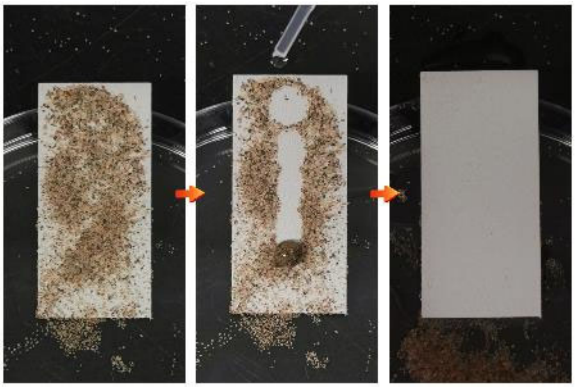

Figure S9. Self-cleaning behavior of the SPET/FD-POS@SiO_2 coating. $V_{non\text{-}solvent}$ = 1.7 mL, m_{SPET} = 2.4 g.

Movie S1. Water droplet freezing process on the Al alloy plate.

Movie S2. Water droplet freezing process on the SPET/FD-POS@SiO_2 coated Al alloy plate. $V_{non\text{-}solvent}$ = 1.7 mL, m_{SPET} = 2.4 g.
